# Two Cases of Benign Biliary Stricture in Young Men

**DOI:** 10.7759/cureus.110151

**Published:** 2026-06-02

**Authors:** Ravi Maharaj, Barry Raghunanan, Rebecca Jardine, Keshan Ramnarace

**Affiliations:** 1 Surgery, The University of the West Indies, St. Augustine, TTO; 2 Surgery, Eric Williams Medical Sciences Complex, Port of Spain, TTO; 3 Medicine and Surgery, The University of the West Indies, Mount Hope, TTO; 4 Medicine and Surgery, The University of the West Indies, St. Augustine, TTO

**Keywords:** benign biliary strictures, biliary strictures, biliary surgery, hepaticojejunostomy, idiopathic biliary disease

## Abstract

Benign biliary strictures are an uncommon cause of obstructive jaundice in young adults, with idiopathic cases requiring surgical intervention being particularly rare. This report presents two young male patients with benign biliary strictures who presented with short histories of jaundice and radiological evidence of biliary obstruction.

The first case involved a 27-year-old man with four months of progressive jaundice, conjugated hyperbilirubinemia, and transaminitis. Imaging demonstrated proximal common bile duct/common hepatic duct obstruction with stricture. Endoscopic retrograde cholangiopancreatography confirmed a tight common hepatic duct stricture, with stenting performed. Definitive management included bile duct excision and Roux-en-Y hepaticojejunostomy. Histopathology showed chronic inflammation and scar tissue without malignancy.

The second case involved a 28-year-old man presenting with a two-week history of jaundice, pruritus, dark urine, and pale stools, with laboratory evidence of conjugated hyperbilirubinemia and transaminitis. Imaging revealed hilar obstruction with intrahepatic and extrahepatic biliary dilatation. Following percutaneous biliary drainage, the patient underwent cholecystectomy, bile duct excision, and Roux-en-Y hepaticojejunostomy. Histology demonstrated mild fibrosis and chronic nonspecific inflammation without malignancy.

These cases highlight that while endoscopic management has become increasingly common for benign biliary strictures, open surgical reconstruction remains a safe, reliable, and definitive option in resource-limited settings, providing durable long-term biliary drainage and diagnostic certainty.

## Introduction

The management of biliary strictures has evolved over the years. The mainstay for malignant etiologies has remained surgery; however, more conservative methods are routinely employed for benign diagnoses. Moreover, there still remains a role for surgical intervention in benign cases. Surgical intervention may be required when malignancy cannot be excluded or when advanced endoscopic expertise is unavailable. While endoscopic techniques (endoscopic retrograde cholangiopancreatography (ERCP)) with stents are typically used to manage benign strictures, surgical intervention is warranted for failed or refractory cases [1].

We present two cases requiring major biliary surgery and reconstruction for benign disease. The aims are to highlight the efficacy of such methods in a low-resource setting and to emphasize their continued role in benign biliary disease.

## Case presentation

Case 1

AB, a 27-year-old man, was referred to the surgical clinic for jaundice. The patient was previously well with no comorbidities but noted gradual-onset icterus for four months. He had no history of biliary colic, sickle cell disease (SCD), or drug use. Initial examination findings were unremarkable except for the scleral icterus. Initial investigations including hematological, biochemical, renal, and liver function and human immunodeficiency virus (HIV), hepatitis, and dengue titers were performed. Results yielded conjugated hyperbilirubinemia with transaminitis, with other assessments returning normal values. Carcinoembryonic antigen (CEA) and carbohydrate antigen 19-9 (CA 19-9) were also negative. Unfortunately, no facilities for IgG4 were available for assessment. The patient went on to have a magnetic resonance cholangiopancreatography (MRCP) two days after admission, which noted a proximal common bile duct/common hepatic duct obstruction with possible stricture. No gallstones were noted. Subsequent ERCP one day after MRCP noted a tight, irregular common hepatic duct stricture proximal to the cystic duct (Figure 1). Brushings were done, and a 7 cm 7-Fr Boston stent was placed with drainage, with the cytology report showing an inadequate specimen for analysis. Multidisciplinary team (MDT) discussions were held regarding the diagnosis and management of the patient. The patient underwent bile duct excision and Roux-en-Y hepaticojejunostomy two days afterwards. No complications were noted during surgical intervention, and no evidence of metastatic disease was noted. The postoperative period was uneventful with improved liver enzymes. Final histology of the gallbladder and common bile duct noted negative margins, but tissue analysis revealed scar tissue and active chronic inflammation of the common bile duct consistent with benign stricture and no evidence of malignancy. After six months of follow-up, the patient remained asymptomatic and anicteric.

**Figure 1:**
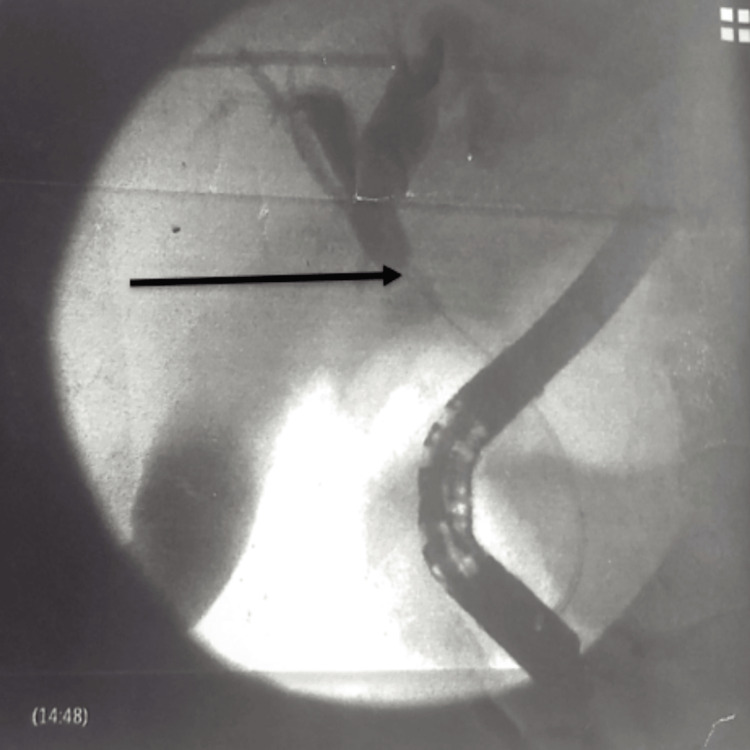
Endoscopic retrograde cholangiopancreatography highlighting the common hepatic duct stricture proximal to the cystic duct (black arrow)

Case 2

DG, a 28-year-old man, was referred to surgery for urgent admission and biliary stent insertion for severe laboratory-confirmed jaundice with radiological evidence of hilar obstruction with possible stricture. The patient had no comorbidities but noted rapidly progressing icterus, pruritus, dark urine, and pale stool for two weeks. Initial examination findings for jaundice noted scleral icterus. No fever or right upper quadrant tenderness was noted. The patient had no history of biliary colic, blood dyscrasias, or drug use. Initial investigations, including hematological, biochemical, renal, and liver function, with HIV, hepatitis, and dengue titers returned negative results. Investigations noted primarily conjugated hyperbilirubinemia and transaminitis with elevated alkaline phosphatase (ALP), while other assessments were unremarkable. Abdominal ultrasound scan (USS) displayed a contracted gallbladder with no evidence of cholelithiasis. Computed tomography of the abdomen and pelvis (CTAP) with contrast noted intrahepatic and extrahepatic (common hepatic) biliary ductal dilation with no focal lesions. The patient went on to have an MRCP the next day which noted not only intrahepatic dilation at the convergence of the right and left intrahepatic ducts at the level of the hilum with obstruction at this level but also the inability to visualize the cystic duct, an empty contracted gallbladder, and no intraductal filling defects or stones (Figure 2). A percutaneous transhepatic catheter (PTC) was placed on the same day, and fluid cytology showed no evidence of malignancy. CTAP with contrast one day post-PTC showed some persistent but improved intrahepatic biliary ductal dilation and mildly improved hyperbilirubinemia. The lack of an identifiable mass, the absence of lymph nodes, the abrupt cutoff at the confluence, and the patient's age favored a benign stricture. The patient subsequently underwent cholecystectomy, bile duct excision, and Roux-en-Y hepaticojejunostomy within 48 hours after CTAP. No complications were noted during the surgical intervention, and no evidence of metastatic disease was noted. The postoperative period was uneventful with normalizing liver function panel. Final histology of the gallbladder and common bile duct yielded negative margins with mild fibrosis and chronic nonspecific inflammation of the common bile duct and denied any evidence of malignancy. Unfortunately, facilities for IgG4 were unavailable for testing. A segment of the jejunum showed focal nonspecific mucosal ulceration. The patient remained asymptomatic and anicteric after six months of routine follow-up.

**Figure 2:**
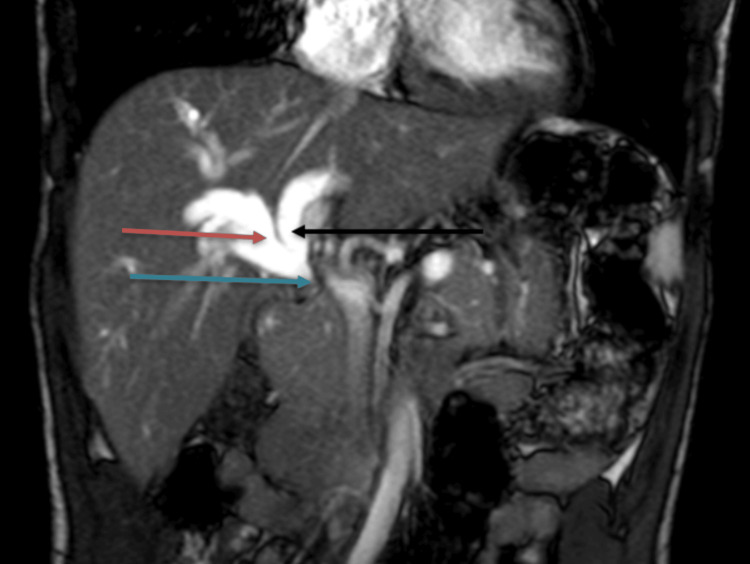
Magnetic resonance cholangiopancreatography showing the biliary stricture (blue arrow), right hepatic duct (orange arrow), and left hepatic duct (black arrow)

Table 1 shows the admission and day 5 postoperative labs for both patients.

**Table 1:** Both patients' admission and day-5 postoperative labs AST: aspartate aminotransferase; ALT: alanine transaminase; CA 19-9: carbohydrate antigen 19-9; CEA: carcinoembryonic antigen; CRP: C-reactive protein; PT: prothrombin time; PTT: partial thromboplastin time; INR: international normalized ratio

Lab parameters	Range	Case 1 (AB)		Case 2 (DG)	
		Preoperative (admission)	Day 5 postoperative	Preoperative (admission)	Day 5 postoperative
Hemoglobin (g/dL)	11-15.5	12.2	13.7	11.2	10.6
White blood cell (10^3^/uL)	4-11.5	8	11	11.8	9.7
Platelets (10^3^/uL)	150-450	240	152	581	449
AST (U/L)	5-45	106	50	159	159
ALT (U/L)	4-48	62	49	116	82
Alkaline phosphatase (U/L)	38-151	122	85	1099	231
Total bilirubin (mg/dL)	0.1-1.5	2.3	1	25.2	4.4
Direct bilirubin (mg/dL)	0-0.8	2.3	0	14.4	0.7
Indirect bilirubin (mg/dL)	0-0.9	0	1	10.8	3.7
Albumin (g/dL)	3.5-5.6	4.5	4.2	4	3.7
Creatinine (mg/dL)	0.2-1.3	1.2	1.1	0.75	0.6
Sodium (mmol/L)	135-145	144	136	142	136
Potassium (mmol/L)	3.6-5.1	4.5	3.8	3.8	4.1
Chloride (mmol/L)	92-108	100	99	102	98
CA 19-9 (U/mL)	0-40	5.8	-	6.9	-
CEA (ng/mL)	0-4	2.1	-	1.9	-
CRP (mg/L)	0-10	15.6	6.3	18.2	5
PT (s)	11-14	12.1	-	12.1	-
PTT (s)	26-31	30	-	31.5	-
INR	1-1.5	0.983	-	0.984	-

## Discussion

Stricture of the bile duct can occur congenitally or from acquired means. Acquired strictures are more often malignant than benign; however, up to 25% of malignant resections would be for benign cases [2-4]. The etiology of biliary stricture is expansive and encompasses inflammatory, infective, malignant, and iatrogenic causes. However, with at least 70% of biliary strictures being malignant, it is paramount that malignancy be accurately ruled out in the case of benign biliary stricture [4]. We seek to look at the current best practice for identifying the cause of biliary stricture and compare that to available methods in resource-limited settings.

Malignant etiologies of biliary stricture include pancreatic cancer, with 70% of pancreatic cancer patients presenting with stricture at initial diagnosis, followed by cholangiocarcinoma. Less common malignant causes include metastatic cancer of the liver, ampullary tumors growing into the bile duct, gallbladder cancer obstructing the bile duct, or malignant periportal lymph nodes. Benign causes in order of frequency include iatrogenic (post-liver transplant or cholecystectomy), primary sclerosing cholangitis, autoimmune diseases (pancreatitis or IgG4-related cholangitis), chronic pancreatitis, infection (HIV cholangiopathy, recurrent pyogenic cholangitis), Mirizzi syndrome, ischemic cholangiopathy, and idiopathic benign biliary stricture [4,5].

The extent of ductal infiltration aids in radiologically distinguishing the malignant or benign nature of the stricture, but this still proves to be difficult. Baskin-Bey et al. noted the use of the Bismuth-Corlette classification; type I and II strictures are more often associated with idiopathic benign strictures and choledocholithiasis than malignant etiology. AB follows this principle and is classified as type II, but DG is classified as type IV, which emphasizes the difficulty in diagnosing benign biliary disease without surgical intervention. This study also noted the gender prevalence for idiopathic benign strictures to be significant (p<0.01), occurring twice as commonly in women. Our study assessed two men, which allows more data to be available on this rare cause [6].

The diagnostic approach involves detailed history and physical examination, imaging, liver tests, tumor markers, ERCP brushings/biopsy, cholangioscopy, endoscopic ultrasound (EUS), PTC, multidisciplinary review, and surgery when uncertainty persists. Thomasset et al., in a retrospective cohort study, highlighted the laboratory tests most commonly associated with a primary hepatopancreatobiliary (HPB) malignancy. Eight hundred and thirty patients were assessed, and it was noted that patients with a normal bilirubin level but deranged liver enzymes (ALP and/or alanine transaminase (ALT)) are more likely to have malignant disease [7].

The difficulty in assessing the benign or malignant nature of these strictures continues to be significant. Cases AB and DG both presented with obstructive jaundice demonstrating the "malignant masquerade" identified by Corvera et al. [8]. In a study of 275 patients with proximal biliary strictures and an unremarkable history of trauma or earlier biliary operation, this study notes 22 cases of benign stricture with no preoperative, clinical, or radiographic features that could reliably distinguish benign strictures from cancerous ones. This is made more difficult when the site and extent of the stricture are displayed (as seen in AB and DG), but the underlying etiology remains unclear due to the absence of stones, mass, adenopathy, or the more diffuse biliary changes of primary sclerosing cholangitis [6,8]. Both AB and DG presented with conjugated hyperbilirubinemia and transaminitis, with DG also presenting with elevated ALP and gamma-glutamyl transferase (GGT). Consequently, both patients were treated as an underlying malignancy was present as a result of a benign etiology not definitively identifiable [4,8].

CA 19-9 cannot be used to reliably determine the etiology of a biliary stricture. Although it is typically associated with pancreatic cancer, it can also be elevated with any cause of biliary obstruction, including cholangiocarcinoma, cholestasis, cholangitis, cirrhosis, and pancreatitis [9]. CEA is even less sensitive (30-86% vs. 80%) and specific (75-95% vs. 80-90%) than CA 19-9. CEA is associated with a wide range of pathologies and cannot definitively detect cholangiocarcinoma [10].

It should be noted that MDT management is paramount in the successful outcomes of patients, especially those with strictures of unknown etiology. Methods of investigation include MRCP, ERCP, and EUS. Their efficacy and sensitivity in diagnosing the cause of the stricture vary greatly and may require a prolonged period of clinical surveillance for the exclusion of underlying occult malignancy. This proves difficult in a resource-limited setting where a definitive surgical procedure can be diagnostic and therapeutic by allowing early and accurate diagnosis via histopathology. CT and MRCP localize pathology and provide information on the characteristics of the biliary stricture while providing a guide for therapeutic ERCP and/or endoscopic intraductal ultrasound (IDUS)-guided fine-needle aspiration (FNA) or biopsy that are used to establish a histological diagnosis. A meta-analysis has shown the low sensitivity and high specificity with ERCP for the diagnosis of malignant biliary stricture with 45% and 99%, respectively, for brush cytology and 48.1% and 99.2%, respectively, for trans-papillary forceps biopsy [11]. Other adjuvant technologies useful in the assessment of the etiology of biliary strictures include free DNA profiling via fluorescence in situ hybridization (FISH) and methionyl-tRNA synthetase 1 (MARS 1), cholangioscopy with biopsy, IDUS, and confocal laser endomicroscopy. These procedures are revolutionizing the field of tissue biopsy but require additional training for providers and are associated with high risk for complications, particularly cholangitis, risk of tumor seeding, and tract recurrences. These advances improve sensitivity to otherwise indeterminate causes but require further studies [12]. IDUS requires expert training, but when used with ERCP, it improves diagnostic accuracy by 11% [13]. Negative biopsies and brushings, therefore, do not exclude bile duct cancers and are frequently falsely negative [8]. Additionally, contemporary approaches such as combined brush cytology plus fluoroscopic biopsy, cholangioscopy-guided biopsy, and EUS should be performed where available to aid diagnostic accuracy.

The surgical resection of the common bile duct and gallbladder via Roux-en-Y hepaticojejunostomy prevents missing an occult malignancy that can occur due to the low sensitivity of endoscopic sampling. The diagnosis of benign biliary stricture among both cases was obtained by excluding the other benign and malignant causes by radiology and more definitively by histology post-excision of the bile duct and gallbladder. Histopathology often defines the major difference in the management of bile duct stricture. Histologic examination of the specimens can identify various benign processes: lymphoplasmacytic sclerosing pancreatitis and cholangitis, primary sclerosing cholangitis, granulomatous disease, nonspecific fibrosis/inflammation, and stone disease. Findings of malignancy would often shunt a patient to the HPB surgeon for definitive surgery. Nondiagnostic findings or benign reports often place the patient for interventional management. It is often difficult to obtain pathologic confirmation, and in most cases, as seen in AB and DG, patients are offered surgical therapy based on clinical suspicion and radiologic evidence. Of note, cholangiocarcinoma was definitively excluded upon histopathological diagnosis for both of our cases.

Management for symptomatic biliary strictures can be endoscopic, interventional, or surgical. Treatment aims to preserve liver function while maintaining long-term drainage and relieving the symptoms of biliary obstruction. Due to advances in endoscopic intervention, it has become the mainstay of benign biliary stricture treatment. Endoscopic intervention has greatly improved with strictures being managed by balloon or bougie dilatation that is followed by the placement of one or more plastic stents or a fully covered self-expandable metal stent (FCSEMS), all of which decrease stricture recurrence. These minimally invasive options have gained favor, showing results which may be equal to surgery with decreased morbidity and faster recovery [13-15]. However, these are not without drawbacks, with risk of needle tract seeding from EUS-FNA, post-ERCP pancreatitis, and the morbidity caused by stent dysfunction resulting in recurrent jaundice and cholangitis. In addition, this may not be feasible in a low-resource environment due to the need for multiple procedures for stenting or replacement and the requirement for specialist training. In our cases, endoscopic therapy was not continued for AB due to the unavailability of the resource for further management. Similarly, for DG, surgical intervention was utilized as a result of a lack of availability of endoscopic equipment. Endoscopic accessibility continues to be a limitation in a low- to middle-income setting.

Further improvements to the minimally invasive management have occurred. Intraductal bipolar radiofrequency ablation (RFA), which is primarily used for malignant strictures, showed immediate stricture improvements in all patients with benign biliary stricture in a 2014 small study. RFA was delivered to nine patients at a power of 10W for 90 seconds per stricture segment followed by balloon dilation with or without stenting. This was not without complications as one patient had stricture relapse after the initial resolution, one required surgery, and one patient had mild post-ERCP pancreatitis [16].

Percutaneous placement of biodegradable biliary self-expanding stents presents a new feasible and effective option in treating benign biliary stenosis that is free from major complications. A 10-person 2012 study found that no stent was visible at the six-month follow-up, which showed neither re-stenosis nor dilatation of the biliary tree; however, three patients experienced episodes of cholangitis [17].

Percutaneous transhepatic biliary drainage (PTBD) is used in cases of failed ERCP, and the surgically altered anatomy approach is less invasive and lower risk compared to surgery. Lee et al. showed a 5% recurrence rate with a 66% success rate. Notably, unsuccessful patients required significantly more procedures and indwelling stents significantly longer than patients whose strictures were resolved by balloon dilation [18].

Balloon enteroscopy-assisted ERCP may improve endoscopic access to the major papilla in patients with surgically altered anatomy, such as Roux-en-Y hepaticojejunostomy, gastric bypass, or following Whipple's procedures [19]. A systematic review and meta-analysis of single balloon enteroscopy (SBE)-assisted ERCP in patients with surgically altered gastrointestinal (GI) anatomy showed procedural success rates of 80.9% when 15 trials assessed for SBE to reach the duodenal papilla, with 69.4% diagnostic success by obtaining a cholangiogram and 61.7% procedural success via biliary intervention. Adverse events occurred in 6.5% of procedures, including pancreatitis (2.2%), perforation (0.8%), and major bleeding (0.4%) [19].

In addition, the management has shifted to the gastroenterologist and left the most complex cases to the surgeons.

Surgical alternative repair of biliary strictures depends on extent, location, access to less invasive methods, and whether strictures are refractory to endoscopic interventions. These include bile duct exploration, Roux-en-Y hepaticojejunostomy, choledochojejunostomy, excision of strictures with end-to-end repair, and choledoduodenostomy.

Although there is an increased risk of morbidity when compared to its less invasive endoscopic counterparts, the Roux-en-Y hepaticojejunostomy is safe and durable, with a success rate of 80-99% [14].

Due to its wide utility and broad applications across different surgical fields, newly trained surgeons are comfortable and experienced with this technique versus the specialty-specific training of the less invasive methods, which would not be as available in a low-resource setting. It is important to note that surgery may be appropriate when malignancy cannot be excluded or when advanced endoscopic options are unavailable. Nevertheless, surgical intervention offers the best long-term results, reducing the chance of recurrent stone formation, the number of endoscopic procedures, and possibly recurrent cholangitis. Although high upfront costs exist, overall costs are decreased when compared to the need for repeated interventions.

A retrospective analysis of Nationwide Inpatient Sample discharges (2004-2011) identified 67160 weighted admissions with surgical biliary duct reconstruction, highlighting the common practice of surgical repair despite the availability of minimally invasive and endoscopic techniques. The majority of cases treated non-malignant causes and displayed a death rate of 4.2% overall and 3% for non-malignant biliary disease not caused by biliary trauma or injury. The overall rate of inpatient death decreased over time to 3.8% (p=0.0148) in 2010-2011. Factors increasing the likelihood of mortality were identified, including admissions that lasted over two weeks, biliary injury or trauma versus other non-malignant disease, malignant disease versus non-malignant disease, revision of biliary anastomosis versus choledochoenterostomy, age greater than 40, and postoperative complications. At least one postoperative complication occurred in 32.3% of patients, including most commonly postoperative infection (13.4%), acute renal failure, operative bleeding (4.7%), GI bleed, deep vein thrombosis (DVT)/pulmonary embolism (PE) (1%), acute myocardial infarction (MI) (1.3%), and acute liver failure [20].

Khalid et al., via a prospective study, reviewed open choledochoduodenostomy on 54 consecutive patients over a nine-year period and showed the effectiveness of the procedure. The overall hospital morbidity was 13% with zero mortality. Follow-up continued for 7.8 years without malignant or disease recurrence, and 96.3% of patients had "good" or "fair" results [21].

Seventy-nine patients who underwent biliary-enteric anastomosis were retrospectively reviewed over 21 years. Forty-three percent underwent a hepaticojejunostomy, 24% choledochojejunostomy, and 33% choledochoduodenostomy. Early complications occurred in 49% of patients: 41% had local complications, and 25% had systemic complications. Complications were wound infection (23%) and bile leak (10%). The mortality rate was 5% [22].

In a retrospective review of patients who had undergone biliary-enteric anastomosis for benign diseases 2-32 years prior, looking at 1003 patients, 63% females showed the rate of cholangiocarcinoma to directly relate to that of cholangitis occurring in the three groups: choledochoduodenostomy patients at 7.6%, trans-duodenal sphincteroplasty patients at 5.8%, and hepaticojejunostomy patients at 1.9%. The Roux-en-Y technique is less prone to intestinal bacterial backflow because of the protective action exerted by the interposed jejunal loop, is exempt from pancreatic reflux unlike the other methods, and thus produces lower rates of cholangitis. The significantly lower rate of cholangiocarcinoma detected in the hepaticojejunostomy group emphasizes the connection between cholangitis and the development of malignancies of the biliary tract and the need for patients experiencing relapsing cholangitis to be monitored for the late development of cholangiocarcinoma [23].

Hepaticojejunostomy for benign lesions was reviewed via a retrospective analysis carried out for 51 patients, of which 68% were males with an average age of 48±13. Its long-term results showed its continued use as a safe and reliable method for the treatment of benign lesions of the bile ducts, even in young patients in need of a long-term biliary bypass. Postoperative complications occurred in 33% of patients with a hospital stay ranging from eight to 90 days, averaging at 24±17 days; reoperation was required for four patients. A mortality rate of 2% was seen, with one death. Long-term complications occurred in 12 patients, including cholangitis in seven patients with stenosis occurring concomitantly in three cases requiring reoperation. When reassessed by a questionnaire at an average of 7.6 years after the operation, 88% of patients were in good or very good condition [24].

The two young male patients presented both with idiopathic benign biliary disease definitively diagnosed by histopathology post-surgical intervention. Major limitations include only two cases, incomplete etiological workup, absence of advanced endoscopic tissue diagnosis, limited histopathological detail, and short follow-up. However, no complications occurred including the notable absence of leakage or cholangitis which are severe potential complications. This ultimately highlights the safety of the procedure in experienced hands. Both patients remained asymptomatic and anicteric several months post-procedure, proving Roux-en-Y hepaticojejunostomy to be a reliable method for the treatment of patients in need of a long-term biliary bypass.

## Conclusions

Benign biliary strictures occur from a variety of causes, but occasionally, they are in fact idiopathic. Open surgery may be a safe and effective option in selected patients, but this case series cannot establish it as preferable to endoscopic management. In areas where surgical specialties are present, it can be the method of choice over more limited, resource-heavy procedures.
